# AI-Assisted Screening of Oral Potentially Malignant Disorders Using Smartphone-Based Photographic Images

**DOI:** 10.3390/cancers15164120

**Published:** 2023-08-16

**Authors:** Vivek Talwar, Pragya Singh, Nirza Mukhia, Anupama Shetty, Praveen Birur, Karishma M. Desai, Chinnababu Sunkavalli, Konala S. Varma, Ramanathan Sethuraman, C. V. Jawahar, P. K. Vinod

**Affiliations:** 1 CVIT, International Institute of Information Technology, Hyderabad 500032, India; vivek.talwar@research.iiit.ac.in (V.T.); jawahar@iiit.ac.in (C.V.J.); 2 INAI, International Institute of Information Technology, Hyderabad 500032, India; pragya.singh@iiit.ac.in (P.S.); varma.konala@iiit.ac.in (K.S.V.); 3 Department of Oral Medicine and Radiology, KLE Society’s Institute of Dental Sciences, Bengaluru 560022, India; nirzamukhia@gmail.com (N.M.); praveen.birur@biocon.com (P.B.); 4 Biocon Foundation, Bengaluru 560100, India; anupama.shetty101@biocon.com; 5 iHUB-Data, International Institute of Information Technology, Hyderabad 500032, India; karishma.desai@ihub-data.iiit.ac.in; 6 Grace Cancer Foundation, Hyderabad 501505, India; chinna@gracecancerfoundation.org; 7 Intel Technology India Private Limited, Bengaluru, India; ramanathan.sethuraman@intel.com; 8 CCNSB, International Institute of Information Technology, Hyderabad 500032, India

**Keywords:** oral cancer screening, deep learning, photograph, smartphone, point-of-care solution

## Abstract

**Simple Summary:**

The early detection of oral cancer is essential for improving patient outcomes. A conventional oral examination by specialists is the clinical standard for detecting oral lesions. However, many high-risk individuals in middle- and low-income countries lack access to specialists. Therefore, there is a need to develop an easy-to-use, non-invasive oral screening tool that enhances the existing system for detecting precancerous lesions. This study explores artificial intelligence (AI)-based techniques to identify precancerous lesions using photographic images of oral cavities in the Indian population. The high performance of deep learning models suggests that an AI-based solution can be deployed for community screening programs in low-resource settings after further improvement and validation.

**Abstract:**

The prevalence of oral potentially malignant disorders (OPMDs) and oral cancer is surging in low- and middle-income countries. A lack of resources for population screening in remote locations delays the detection of these lesions in the early stages and contributes to higher mortality and a poor quality of life. Digital imaging and artificial intelligence (AI) are promising tools for cancer screening. This study aimed to evaluate the utility of AI-based techniques for detecting OPMDs in the Indian population using photographic images of oral cavities captured using a smartphone. A dataset comprising 1120 suspicious and 1058 non-suspicious oral cavity photographic images taken by trained front-line healthcare workers (FHWs) was used for evaluating the performance of different deep learning models based on convolution (DenseNets) and Transformer (Swin) architectures. The best-performing model was also tested on an additional independent test set comprising 440 photographic images taken by untrained FHWs (set I). DenseNet201 and Swin Transformer (base) models show high classification performance with an F1-score of 0.84 (CI 0.79–0.89) and 0.83 (CI 0.78–0.88) on the internal test set, respectively. However, the performance of models decreases on test set I, which has considerable variation in the image quality, with the best F1-score of 0.73 (CI 0.67–0.78) obtained using DenseNet201. The proposed AI model has the potential to identify suspicious and non-suspicious oral lesions using photographic images. This simplified image-based AI solution can assist in screening, early detection, and prompt referral for OPMDs.

## 1. Introduction

Oral potentially malignant disorders (OPMDs) are a set of disorders that exhibit an increased risk of malignant transformation [1]. These lesions present an array of clinical variations, including white, red, or mixed red-white lesions with verrucous, papillary, corrugated, atrophic, and ulcerated presentations [1]. In addition, lesions like frictional keratosis, chemical injury, leukoedema, candidiasis, denture-associated stomatitis, and desquamative or autoimmune disorders exhibit overlapping clinical features, making the diagnosis of OPMDs challenging [1,2,3]. Though oral cancers can develop de novo, OPMDs share numerous risk factors and molecular/genetic alterations with oral cancers [2,3]. Studies indicate that most habit-associated oral cancers evolve from pre-existing OPMDs. Preliminary epidemiological research and systematic reviews report that 0.1 to 40% of leukoplakia develops into oral cancer [3,4,5,6]. Hence, the early diagnosis and differentiation of OPMDs from clinically similar-appearing lesions are vital for limiting the possible malignant change and improving treatment outcomes.

The oral cavity can be easily visualized without special instruments compared to other internal organs. A Conventional Oral Examination (COE), which involves a visual inspection by a specialist, is the clinical standard for detecting oral lesions [7]. The clinical assessment of OPMDs is subjective, and biopsies remain the gold standard for their definitive diagnoses. However, many high-risk individuals in low- and middle-income countries lack access to specialists or adequate health services, leading to delays in diagnoses and referrals for patients with OPMDs and oral cancer [8]. On the other hand, diagnoses based on biopsies are not ideal for screening due to their invasive nature and limited availability of experts at point-of-care or remote locations. Therefore, there is a definite need to develop an easy-to-use, non-invasive oral screening tool that enhances the existing system for managing OPMDs. Comprehensive clinical assessments, swift patient referrals for biopsies, and the cessation of habits/risk factors are keys to better patient care.

Different studies have evaluated autofluorescence imaging devices as clinical adjuncts to COE for detecting OPMDs and oral cancer. These studies showed that combining autofluorescence visualization with COE provides better accuracy than either method alone [9]. Multispectral screening devices incorporating different lights (white, violet, and green-amber) have shown promise in maximizing the advantages of white light and fluorescence-based examinations for detecting OPMD [10,11]. An accurate interpretation of results requires training and an understanding of oral pathology. Since an oral examination by an expert is not always feasible in primary care or community settings, implementing an automatic classification system based on oral cavity imaging would be beneficial. Increasing evidence shows that deep learning techniques can match or surpass human experts in diverse prediction tasks, including classifying different cancers and detecting diabetic retinopathy [12]. Artificial intelligence (AI) in healthcare is poised to improve the experience of both clinicians and patients.

In this study, we examined the potential of AI in detecting OPMDs from the photographic images of oral cavities in the Indian population. A large dataset of oral cavity images captured using a regular smartphone camera from the community screening camps in India was used for this purpose. This dataset comprises photographic images of normal oral cavities, OPMDs, and a smaller set of oral cancer images. The major objective is to evaluate the performance of different state-of-the-art deep learning models to identify suspicious lesions comprising of OPMDs and oral cancer using white light imaging. Convolutional Neural Networks (CNNs) are well-known deep learning architectures widely used in image classification tasks, including in the medical domain, for identifying various diseases [13,14,15]. The success of transformers in natural language processing has led to their adaptation to computer vision problems. Dosovitskiy et al. (2021) showed that vision transformers, self-attention-based architectures, can attain excellent performance compared to CNNs in various imaging benchmarks, requiring fewer computational resources to train [16]. A comparison of the performance of deep learning models using convolution and transformer architectures was performed with independent test sets. Implementing the best-performing models on a smartphone-based point-of-care platform may help the community screening program, especially in resource-limited settings.

## 2. Methods

### 2.1. Dataset Description

**Figure 1:**
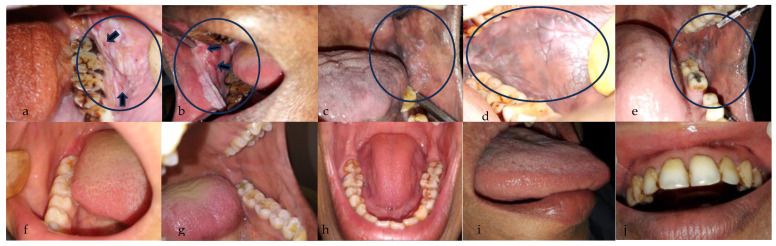
Intraoral images of suspicious and non-suspicious lesions. (**a**,**b**): Buccal mucosa showing a white lesion with few red areas (indicated by arrows) suggestive of non-homogenous leukoplakia. (**c**): Left buccal mucosa showing a reticular, lacy white lesion suggestive of lichen planus. (**d**,**e**): Left buccal mucosa showing a white patch suggestive of homogenous leukoplakia. (**f**,**g**): Normal appearance of left and right buccal mucosa. (**h**,**i**): Dorsal and lateral surface of a tongue showing no abnormalities. (**j**) Upper labial mucosa and vestibule showing no abnormalities. The black circle indicates the region of interest.

Intraoral smartphone-based images (white light images) were retrieved from the database of community-based outreach programs for the early detection and prevention of oral cancer, Biocon Foundation and the Department of Oral Medicine and Radiology, KLE Society’s Institute of Dental Sciences, Bengaluru. The program was reviewed by the institutional review board. The intraoral images were collected by front-line healthcare workers (FHWs), following skill training conducted by oral medicine specialists. The training module covered a broad range of knowledge related to the oral cancer disease burden, awareness, early detection, and prevention. The training tools included PowerPoint presentations, focus groups, and an in situ simulation. The in situ simulation involved a chair, a patient, and a step-by-step guide to examining the oral cavity and identifying normal mucosa or tobacco-induced lesions. Before the training, a pre-test (questionnaire) was conducted to determine the baseline knowledge of the participating FHWs. Following the training, a post-test was conducted to assess the effectiveness of the training. If the FHWs failed to score on the questionnaires, they were re-trained. In the end, a clinical manual was handed over to them for reference. In the field setting, the oral cavity (predominantly buccal mucosa) images were captured using a smartphone camera with a minimum resolution of 5 megapixels (Figure 1). The patient’s demographic information and medical and habit history, such as alcohol, tobacco, pan use, and smoking, were collected. The patients included in the study were above 18 years of age. Written informed consent was obtained from all the patients. The quality of the intraoral images collected by the FHWs was evaluated from time to time by the specialist, and if the images were poor, the patients were re-screened.

Three oral medicine specialists provided an image-level annotation and classified them into suspicious and non-suspicious images. The suspicious category majorly includes images of OPMDs as per the standard clinical criteria (homogenous leukoplakia, non-homogenous leukoplakia, erythroplakia, verrucous leukoplakia, oral lichen planus, oral submucous fibrosis, tobacco pouch keratosis) [1]. The dataset also includes a few oral cancer images that are ulceroproliferative or exophytic growths. The non-suspicious category includes images of normal, normal variations, and benign lesions. The specialist diagnosis was used as a reference standard. The reference standard in our study was taken based on the studies conducted by Birur et al. (2019) and (2022), which showed that the remote specialist was as accurate as the onsite specialists in diagnosing OPMDs and oral cancer. The onsite specialist diagnosis showed high sensitivity (94%) compared to histology, while remote showed high accuracy compared with onsite specialists (sensitivity: 95%; specificity: 84%) [17,18,19]. These images from the database were manually checked to exclude those with significant blur, distortions due to flash or out-of-focus, or where the lesions were not visible. After exclusion, the final dataset includes 2178 images, with 1120 suspicious and 1058 non-suspicious images. The final dataset was randomly divided into training, validation, and testing sets comprising 1344, 412, and 422 images, respectively, for the initial testing of various models (Table 1). The performance of the best-performing models was also tested using an additional independent test set comprising 440 photographic images taken by untrained FHWs (set I). Set I contains 220 suspicious and 220 non-suspicious images collected during oral cancer screening camps conducted by Grace cancer foundation at Telangana.

### 2.2. Model Architecture

**Figure 2:**
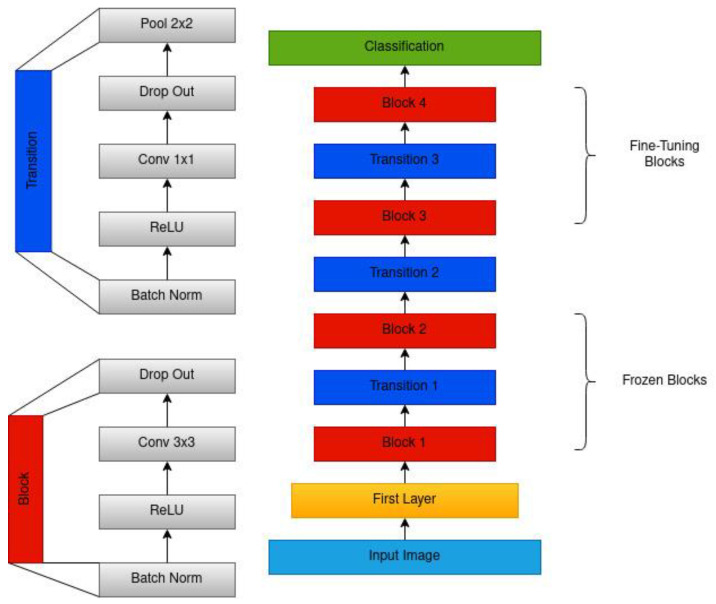
The architecture of the modified DenseNet201. The architecture includes a series of dense blocks, each comprising a convolution layer, ReLU, and batch normalization. Each block is connected to all other blocks in a feed-forward manner. It also uses a transition layer, comprising batch normalization, a convolution layer, and a pooling layer, between each block that helps reduce the spatial dimensionality and number of features.

We focused on training a deep learning model to help identify suspicious lesions from smartphone-based oral cavity images. In this study, we adopted different CNN architectures: VGG19 [20], Inception ResNet-V2 [21], MobileNet-V2 [22], DenseNet-121, DenseNet-169 and DenseNet-201 [23], for identifying suspicious lesions. The architecture of the newly proposed DenseNet201 used for training is shown in Figure 2.

**Figure 3:**
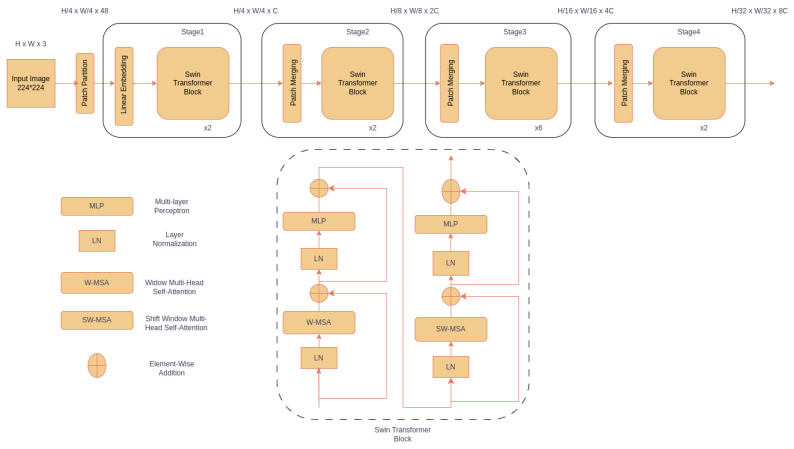
The architecture of the Swin Transformer. The input image is split into non-overlapping patches, and a linear embedding layer is applied to project the raw-valued features to an arbitrary dimension. These are passed through the Swin Transformer block comprising of two multi-head attention (MSA) modules with regular and shifted window configurations (represented as W-MSA and SW-MSA, respectively, in the lower panel). Each module consists of a normalization layer (LN), attention module (W-MSA or SW-MSA), LN, and two-layer multi-layer perceptron (MLP).

We also trained different vision transformers: Vanilla Vision Transformer (ViT) [16], Data-Efficient Image Transformers (DeiT) [24], and Swin Transformers [25] to identify suspicious lesions. The architecture of the Swin Transformer used for training is shown in Figure 3. Swin transformer is a recent vision transformer that can produce hierarchical feature representations and has linear computational complexity related to the input image size [25].

### 2.3. Model Training and Testing

The input images of a 3-to-5-megapixel resolution were resized to 224 × 224 before feeding as an input to CNNs and transformers. CNNs pre-trained on the ImageNet dataset were used for transfer learning. They were fine-tuned by freezing either one-third (VGG19, Inception ResNet-V2, MobileNet-V2) or half (DenseNet family) of the architecture to capture low-level features like edges, lesion size, and colour variation and re-training the remaining part of the network. During the training process, the model was optimized using Adam optimizer with a learning rate of 0.0001, which adjusts network weights based on the error between the predicted and actual output. The model was trained for 50 epochs with a batch size of 16 using categorical cross-entropy loss. The validation loss was used as a criterion for early stopping in case of overfitting. In fine-tuning transformer architectures, a cosineannealing scheduler was used, which includes the warm-up phase with the same learning rate that increases linearly before decreasing according to cosine decay. All the experiments in the paper were implemented in PyTorch (version 2.0.1) on an Nvidia A100 GPU.

### 2.4. Evaluation Metrics

Different metrics were used to compare the performance of models based on convolution and transformer architectures. Metrics were calculated using true positive (TP), true negative (TN), false positive (FP), and false negative (FN) samples.

Precision measures the proportion of the model’s positive predictions that were correct.

(1) $$\;\;\;Precision=\frac{TP}{\left(TP+FP\right)}$$

Recall (sensitivity) measures the proportion of true positive samples correctly identified by the model.

(2) $$\;\;\;\;Recall\;\left(Sensitivity\right)=\frac{TP}{\left(TP+FN\right)}$$

Specificity measures the proportion of true negative samples correctly identified by the model.

(3) $$Specificity=\frac{TN}{\left(TN+FP\right)}$$

The F1-score is the harmonic mean of precision and recall.

(4) $$\;\;\;\;\;F1-score=\frac{2\times \left(Precision\times Recall\right)}{\left(Precision+Recall\right)}$$

AUC measures the area under the receiving operator characteristic (ROC) curve, which shows the plot of the true positive rate (TPR) against the false positive rate (FPR).

(5) $$TPR=\frac{TP}{\left(TP+FN\right)}$$

(6) $$FPR=\frac{FP}{\left(FP+TN\right)}$$

## 3. Results

Six pre-trained CNNs were re-trained on 1344 oral cavity images. These neural networks detect suspicious lesions with different performance levels on the test set (Table 2). The best-performing model was from the DenseNet family (DenseNet201) with precision, recall (sensitivity), specificity, and F1-score of 86%, 85%, 83%, and 86%, respectively. The structure of each of the pre-trained networks was different in terms of the number of layers and size of the input. The important characteristic to consider is the trade-off between performance, speed, and network size. The DenseNet family satisfied these requirements, while VGG19 showed the worst performance. Increasing the size of the DenseNet models led to an improvement in the F1-score (Table 2).

As an alternative to CNN, we also trained vision transformer architectures, which are based on the concept of self-attention. Three variants of Swin Transformers (tiny, small, and base) were compared with ViT and DeiT (Table 3). Swin Transformers yielded better performance with an approximately 10% increase in performance metrics compared to ViT and DeiT. The best precision, recall (sensitivity), specificity, and F1-score results of 86%, 86%, 83%, and 86% were obtained for Swin Transformer (base). Although both DenseNet201 and Swin Transformer (base) yielded comparable best performance, Swin (base) had 88 million parameters compared to the 20 million parameters of DenseNet201 (Table 2 and Table 3).

**Figure 4:**
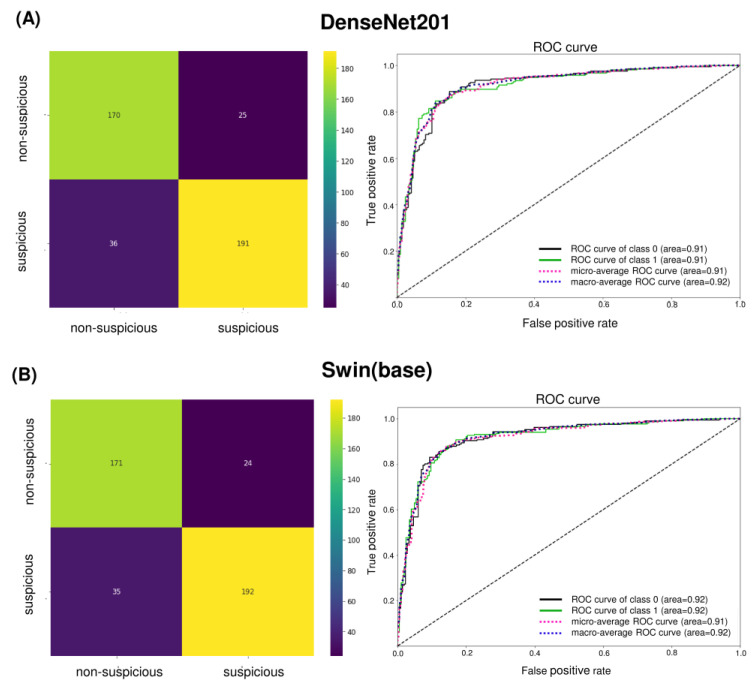
Confusion matrix and ROC curve of (**A**) DenseNet201 and (**B**) Swin Transformer (base). The number of TNs, TPs, FNs, and FPs are given, which are used for computing various performance metrics of models. The ROC curve shows the relationship between false positive and true positive rates and is used for calculating the AUC (area). Class 0 is non-suspicious, and class 1 is suspicious.

**Figure 5:**
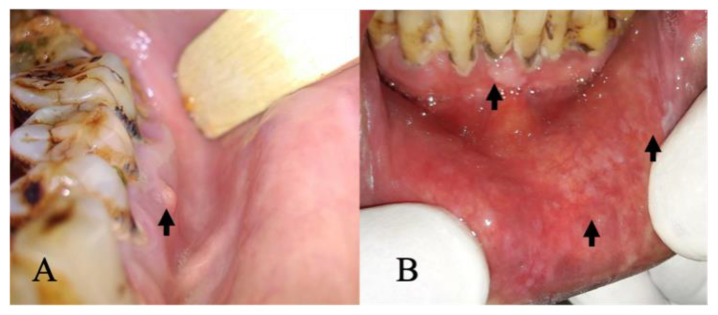
(**A**) False positive: intraoral image showing elevated lesion suggestive of periapical abscess (indicated by arrow). (**B**) False negative: intraoral image showing lower labial mucosa with white areas (indicated by arrow) and periodontitis in the lower anterior region (indicated by arrow).

The confusion matrix of the best-performing models, DenseNet201 and Swin Transformer (base), shows only subtle differences with AUC values greater than 90% for both cases (Figure 4). Clinically, the false positives in the internal test set were primarily those with tobacco stains, physiologic melanosis, aphthous ulcers, and periodontal diseases (gingivitis, abscess, and recession) (Figure 5A). Few discrepancies could be due to lesion localization, topography, or physiologic variations. Amongst false negatives, most lesions diagnosed as non-suspicious included those that presented as early speckled (white-red) areas, gingival desquamation, or traumatic keratosis (Figure 5B). Combined with COE by general dentists, false positives can be easily limited. However, reducing false negatives and enhancing AI sensitivity is critical.

**Figure 6:**
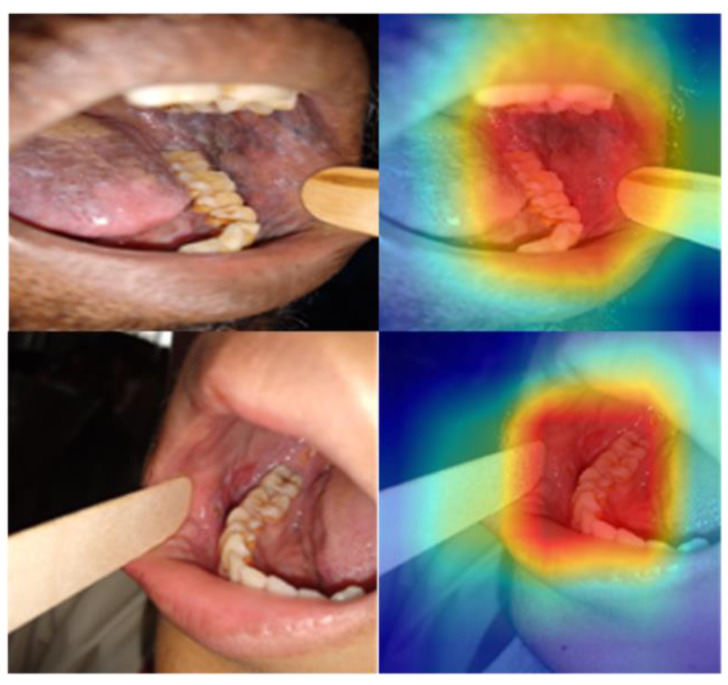
GradCAM visual explanation for the model decision. The colour heatmap highlights the areas in the input image contributing to the decision made by the model, with red regions representing a high score for the class.

Further, the class-activation map was generated using the Gradient-weighted Class Activation Mapping (GradCAM) to provide a visual explanation for the decision [26]. GradGAM shows that the best-performing models (DenseNet 201 and Swin (base)) are focused on the relevant areas for making the prediction (Figure 6). However, the heat map is broader, given that the model is trained on the image-level label, and further improvement is possible with the region-of-interest annotation.

**Figure 7:**
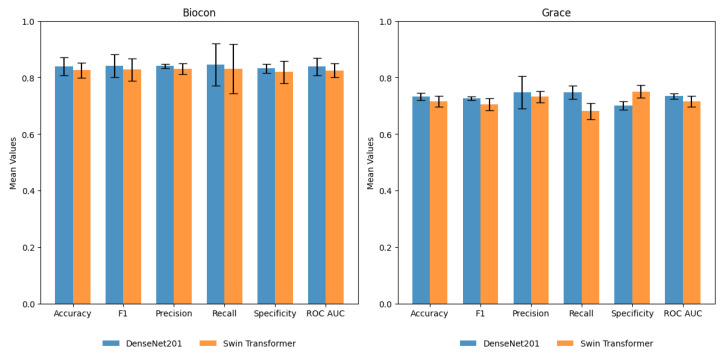
Performance of DenseNet201 and Swin Transformer (base) on the Biocon and Grace test sets. The average value of performance metrics with a 95% confidence interval are shown.

A five-fold cross-validation (CV) of DenseNet201 and Swin Transformer (base) was performed to study the generalizability of models to different train and validation splits. The training and validation data in Table 1 were merged and randomly partitioned into five-fold, maintaining the class balance. The average performance of models on validation (Figure S1) and test sets (Figure 7) is reported with a 95% confidence interval (CI) (Table S1). DenseNet201 yielded the best average F1-score of 0.84 (CI 0.79–0.89), while Swin Transformer (base) model yielded the best average F1-score of 0.83 (CI 0.78–0.88). The Youden Index, which measures the diagnostic test’s ability to balance sensitivity and specificity, was0.71 and 0.67 for DenseNet201 and Swin Transformer (base), respectively. Recall (sensitivity) shows a broader CI than specificity, suggesting uncertainty in detecting suspicious cases. However, it can be noted that a drop in performance is observed in only 1 out of 5 runs of the model, and further data sampling is required to get a more accurate CI estimate.

Further, these models were also tested using an independent test set I (440 images) from the Grace foundation. DenseNet201 yielded an average recall (sensitivity), specificity, and F1-score of 0.75 (CI 0.68–0.82), 0.70 (CI 0.68–0.72), and 0.73 (CI 0.67–0.78), respectively. DenseNet201 showed better performance compared to Swin Transformer on test set I (Figure 7). This set consists of images collected by untrained FHWs with considerable variation in the quality and focus of images. A drastic shift in data quality led to a significant drop in the performance of AI models. Additionally, including a small subset of advanced lesions/oral cancers and differences in lesion localization and surface characteristics affected the FN and FP percentages. On the other hand, the internal test set from Biocon consists of images collected by trained FHWs with consistency in quality and similarity to the training and validation set. The similar data quality led to consistency in the performance of AI models. The results of the independent test set I dictate a need for robust model training. Increasing variations in the dataset can enhance the model’s performance and clinical applicability for the routine screening of OPMDs.

## 4. Discussion

The early detection of oral cancer, particularly the precursor lesions (OPMDs), is essential for improving patient outcomes and quality of life. The present study explored the effectiveness of AI-assisted screening methods for detecting OPMDs using photographic images of oral cavities in the Indian population. A workable solution may lead to the development of a simple yet reliable point-of-care smartphone-based platform that can be used for community screening programs in India. With recent developments in deep learning, it is imperative to study the performance of advanced models to detect suspicious lesions from white light images. Lightweight models with fewer parameters were considered, given that these models can be implemented in computationally limited settings for mobile medical applications in the future. The performance of deep learning models with a high F1-score suggests that the AI-based solution using white light imaging can be deployed for community screening programs after further improvement and validation. The proposed solution includes a simple but methodological approach to capturing images that ensures consistency in lesion position and focal distance and eliminates the need for additional methods using fluorescence devices or light sources.

We showed that the DenseNet201 and Swin (base) Transformer are the best-performing models for detecting suspicious oral lesions using white light images. The best model achieves an average F1-Score of 0.84 on the internal test set. The DenseNet architecture offers several advantages, including mitigating the vanishing gradient problem, supporting feature propagation and reuse, and substantially reducing the number of parameters. By utilizing feature reuse, DenseNet maximizes the network’s potential, resulting in compact models that are both simple to train and highly parameter efficient. In contrast to DenseNet, the Swin Transformer dynamically computes attention weights for each patch, allowing for it to focus on the most informative regions of the image. Each stage of the Swin Transformer involves processing at different levels of abstraction by grouping patches into non-overlapping windows and computing self-attention within each window. By shifting these windows by a fixed amount, the Swin Transformer captures spatially varying patterns in the input image. The resulting features from each stage are then fused using a hierarchical attention mechanism, which captures both local and global contexts. This allows for the Swin Transformer to learn fine-grained and higher-level information, leading to a better performance.

Our proposed solution improves upon the earlier studies that attempt to apply deep learning techniques for identifying OPMDs using different imaging modalities. Uthoff et al. (2018) and Song et al. (2018) combined the dual modalities of autofluorescence and white light imaging captured using the smartphone platform and trained a CNN model (VGG-M) to identify suspicious oral lesions (OPMDs and oral cancer) [27,28]. The model inference was performed on a cloud server, achieving 85% sensitivity and 88% specificity. Song et al. (2021) extended this idea to real-time model inference in resource-limited settings using MobileNet, achieving 79% sensitivity and 82% specificity [29]. The model trained only on white light images showed limited efficiency in these studies. White light imaging only captures the lesion’s surface features, which may appear similar to other disease conditions. Additionally, variations in lighting conditions, camera angles, and other factors can further impact the accuracy and reliability of white light imaging. Recent studies provide further evidence that white light imaging can be potentially useful for detecting OPMDs and oral cancer [30,31,32,33,34,35,36,37,38]. However, most of these studies were carried out on a smaller set of OPMD images, requiring the region-of-interest annotation. The model pipeline included multiple steps involving object detection and classification from oral cavity photographs. The dataset used in these studies comes from different countries (UK, Brazil, China, Thailand) which are not openly available.

The model’s performance with white light imaging alone makes the interpretation simpler than dual modality. The screening solution reduces the expertise level required for imaging, and trained community healthcare workers can be deployed for large-scale screening. The proposed method is validated on a limited number of oral cavity images, and the adoption of this solution requires validation on a larger cohort from different demographics in India. The onsite specialist diagnosis was considered a reference standard given that in a low-resource setting, the compliance of OPMD patients to undergo biopsies is low, which is a significant challenge to overcome in large-scale screening programs. The model’s generalizability to other patient populations and smartphone cameras needs further testing. An evaluation should include diverse profiles of diseases to establish the performance characteristics. Future studies will focus on collecting more oral cavity images and model optimization using a bounding box to specify the region of interest. An annotation step may help since each patient’s oral mucosa may exhibit different colours/pigmentations, and photographs with surrounding teeth, retractors, or other parts of the face can be included. Oral cavity images with the patient’s habit history can be combined to improve the model performance further.

## 5. Conclusions

The present study showed a promising application of AI models in diagnosing OPMDs and cancers using oral cavity images. This AI-based solution could improve screening efficiency, enabling timely intervention and better patient outcomes. We tested the models on multiple independent test sets which indicated the need to train the model on noisy heterogeneous data. The proposed solution is easily deployable since it relies on a lightweight model framework and requires only images captured by a smartphone camera. Future work will focus on streamlining the real-time model inference to help FHWs with referrals for high-risk patients in the remote setting.

## Figures and Tables

**Table 1:** The number of suspicious and non-suspicious images in the train, validation, and test set.

Image Category	Train	Validation	Test	Total
Suspicious	670	216	234	1120
Non-Suspicious	674	206	178	1058
**Total**	**1344**	**412**	**422**	**2178**

**Table 2:** Comparison of convolution-style architectures on the internal test dataset (*n* = 422) in Table 1. Macro-averaged precision, recall (sensitivity), specificity, and F1-score are reported.

Method	Parameters	Precision	Recall (Sensitivity)	F1-Score	Specificity
VGG19	138 M	0.69	0.68	0.68	0.58
InceptionResNet-V2	56 M	0. 72	0.72	0.72	0.72
MobileNet-V2	9.4 M	0.75	0.75	0.75	0.73
DenseNet121	8 M	0.85	0.85	0.85	0.83
DenseNet169	14 M	0.84	0.83	0.84	0.78
**DenseNet201**	**20 M**	**0.86**	**0.85**	**0.86**	**0.83**

**Table 3:** Comparison of transformer-style architectures on the internal test dataset (*n* = 422) in Table 1. Macro-averaged precision, recall (sensitivity), specificity, and F1-score are reported.

Method	Parameters	Precision	Recall (Sensitivity)	F1-Score	Specificity
ViT	86 M	0.77	0.77	0.77	0.77
DeiT	86 M	0.77	0.75	0.75	0.76
Swin (Tiny)	29 M	0.84	0.84	0.84	0.73
Swin (Small)	50 M	0.85	0.85	0.85	0.75
**Swin (Base)**	**88 M**	**0.86**	**0.86**	**0.86**	**0.83**

## Data Availability

The datasets generated during and/or analysed during the current study are not publicly available due to specific institutional requirements governing privacy protection; however, they are available from the authors on reasonable request within the terms of the data use agreement and compliance with ethical and legal requirements. Models for the detection of OPMDs are provided in the Github repository: https://github.com/vision1307/opmd.

## Supplementary Materials

The following supporting information can be downloaded at: https://www.mdpi.com/article/10.3390/cancers15164120/s1. Figure S1: Performance of DenseNet201 and Swin Transformer (base) on the Biocon validation set. The average value of performance metrics with a 95% confidence interval are shown. Table S1: DenseNet201 and Swin Transformer (base) cross-validation performance on the Biocon and grace test sets. The average value of performance metrics with a 95% confidence interval are given in the bracket.
