# Supplementary material for: AI-Assisted Screening of Oral Potentially Malignant Disorders Using Smartphone-Based Photographic Images
Source: Cancers (Basel). 2023 Aug 16;15(16):4120. doi: 10.3390/cancers15164120 (PMC10452422; doi:10.3390/cancers15164120)
Supplement: Supplementary file 1 [file cancers-15-04120-s001.zip › cancers-2451954-supplementary.pdf]

Supplementary information

AI-Assisted Screening of Oral Potentially Malignant Disorders Using Smartphone-Based Photographic Images

Vivek Talwar, Pragya Singh, Nirza Mukhia, Anupama Shetty, Praveen Birur, Karishma M. Desai, Chinnababu Sunkavalli, Konala S. Varma, Ramanathan Sethuraman, C.V. Jawahar and P. K. Vinod

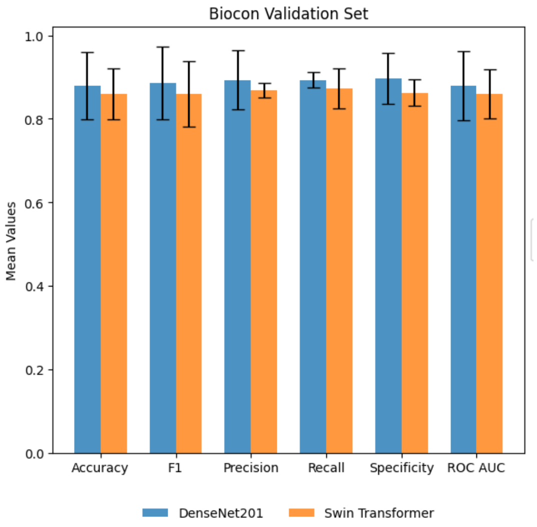

Figure S1: Performance of DenseNet201 and Swin Transformer (base) on the Biocon validation set. The average value of performance metrics with a 95% confidence interval are shown.

Table S1: DenseNet201 and Swin Transformer (base) cross-validation performance on the Biocon and grace test sets. The average value of performance metrics with a 95% confidence interval are given in the bracket.

**Biocon (test)**					
DenseNet201	0.84<br>(CI 0.80-0.88)	0.84<br>(CI 0.83-0.85)	0.85<br>(CI 0.75-0.94)	0.84<br>(CI 0.79-0.89)	0.83<br>(CI 0.81-0.85)
Swin Transformer (Base)	0.83<br>(CI 0.79-0.86)	0.83<br>(CI 0.80-0.85)	0.83<br>(CI 0.72-0.94)	0.83<br>(CI 0.78-0.88)	0.82<br>(CI 0.77-0.87)
**Grace (test)**					
DenseNet201	0.73<br>(CI 0.72-0.75	0.75<br>(CI 0.68-0.82)	0.75<br>(CI 0.68 -0.82)	0.73<br>(CI 0.67-0.78)	0.70<br>(CI 0.68-0.72)
Swin Transformer (Base)	0.72<br>(CI 0.69-0.74)	0.73<br>(CI 0.71-0.76)	0.68<br>(CI 0.65-0.72)	0.70<br>(CI 0.68-0.73)	0.75<br>(CI 0.72-0.78)
